# Short-and long-term effects of ischemic postconditioning in STEMI patients: a meta-analysis

**DOI:** 10.1186/s12944-015-0151-x

**Published:** 2015-11-16

**Authors:** Jing Gao, Junyi Luo, Fen Liu, Yingying Zheng, Bangdang Chen, Qingjie Chen, Yining Yang

**Affiliations:** Department of Cardiology, First Affiliated Hospital of Xinjiang Medical University, Urumqi, 830054 P.R., China; Xinjiang Key Laboratory of Cardiovascular Disease Research, Urumqi, 830054 P.R., China; Department of endocrinology, Fifth Affiliated Hospital of Xinjiang Medical University, Urumqi, 830011 P.R.China

**Keywords:** Ischemic postconditioning, STEMI, Ischemic/reperfusion injury, Cardioprotection, Infarct size

## Abstract

**Background:**

Compelling evidence from large randomized trials demonstrates the salutary effects of ischemic postconditioning on cardioprotection against ischemic/reperfusion injury. However, some studies appear negative findings.

This study was designed to assess the short-and long-term effects of postconditioning (Poc) in studies including evolving ST-elevation myocardial infarction (STEMI).

**Methods:**

Relevant studies were identified through an electronic literature search from the PubMed, Library of Congress, Embase, Cochrane Central Register of Controlled Trials, and ISI Web of Science. Studies published up to December 2014 were eligible for inclusion. Patients older than 18 years presenting within 12 h of the first STEMI and eligible for angioplasty were considered for the study.

**Results:**

The 25 trials allocated 1136 patients to perform locational postconditioning cycles at the onset of reperfusion and 1153 patients to usual percutaneous coronary intervention (PCI). Ischemic postconditioning demonstrated a decrease in serum cardiac enzymes creatine kinase (CK) and CK-MB (*P* < 0.00001 and *P* =0.25, respectively) in the subgroup analysis based on direct stenting. Reduction in infarct size by imaging was showed during7 days after myocardial infarction (*P* =0.01), but not in the longterm (*P* = 0.08). The wall motion score index was improved in both the short term within 7 days (*P* = 0.009) and the long term over 6 months after receiving Poc (*P* = 0.02). All included studies were limited by the high risk of performance and publication bias.

**Conclusions:**

Ischemic postconditioning by brief interruptions of coronary blood flow at the onset of reperfusion after PCI appears to be superior to PCI alone in reducing myocardial injury and improving left ventricular function, especially in patients who have received direct stenting in PCI.

## Background

Ischemic/reperfusion injury appearing after primary percutaneous coronary intervention (PCI) abrogates myocardial salvage and may increase infarct size [1]. It was proved that ischemic preconditioning, which involved a series of brief ischemia/reperfusion cycles and performed before ischemia, was explicitly a cardioprotective strategy [2–4]. Unlike preconditioning, ischemic postconditioning, which involves brief episodes of ischemia/reperfusion during early reperfusion, has been demonstrated to be effective in many studies [5–9]. Ischemic postconditioning has a promising potential to be applied in the clinic. However, some studies suggest that ischemic postconditioning during primary PCI does not reduce infarct size or improve myocardial function recovery [10, 11]. The purpose of this paper was to further summarize the evidence supporting cardioprotection of ischemic postconditioning in patients with acute STEMI by conducting a meta-analysis of the published literature.

## Methods

### Search strategy

To avoid insufficient number of studies, an electronic literature search was simultaneously conducted across the PubMed, Library of Congress, Embase, Cochrane Central Register of Controlled Trials, and ISI Web of Science. Two independent evaluators reviewed all English language articles published up to 2014. The following key words were used as search terms: ischemic postconditioning, reperfusion, ischemic reperfusion injury, primary percutaneous intervention, controlled trials, and randomized controlled trials (RCTs). All prospective, randomized, single-center, or multicenter clinical trials were included.

### Eligibility criteria

Criteria for inclusion were as follows:(1) subjects with explicit STEMI, (2) two reperfusion strategies, PCI with postconditioning (Poc) or conventional (Con) PCI, were compared, (3) a similar baseline between Poc and the control group, with a good match of age, gender, ischemic time, and risk factors, (4) one or more myocardial injury–related indicators, which involve peak creatine kinase (CK), peak CK-MB, ST-segment resolution, infarct size (IS), left ventricular ejection fraction (LVEF), and wall motion score index (WMSI). Continuous variables were reported as mean ± standard deviation (SD). Only the latest study was included in the meta-analysis for identical or largely similar articles.

### Study selection and quality assessment

Two investigators independently assessed the eligibility of identified studies. The studies that were evaluated were RCTs that focused on the role of Poc in STEMI. Published abstracts or without data were excluded. Disagreement resolved by discussion or by referral to a third assess or if necessary. Complete consensus among the authors on the final results was achieved. Studies included in the meta-analysis had to fulfill the aforementioned eligibility criteria. The criteria for study quality outlined by the Cochrane Reviewer’s Handbook 4.2were adopted for quality assessment of included RCTs. These criteria were as follows: (1) correct random methods, (2) randomization, (3) blindness assessment, (4) completeness of the follow-up and using ITT (intention-to-treat) analysis to deal with the dropouts.

### Statistical analysis

Continuous data were reported either as mean (SD) or median (interquartile range). For continuous data, mean difference was calculated where same scale was used to measure relevant outcomes (peak CK, peak CK-MB, LV EF, and WMSI). A random effects model was used to pool data, and the corresponding forest plots were constructed. The Cochran’s Q test was used to assess the heterogeneity among studies and was complemented by the *I*^2^ statistic [12]. All analyses were conducted using the statistical software Review Manager (RevMan) version 5.3. The authors are solely responsible for the design and conduct of this study and its final contents.

## Results

### Identification of studies

**Fig. 1:**
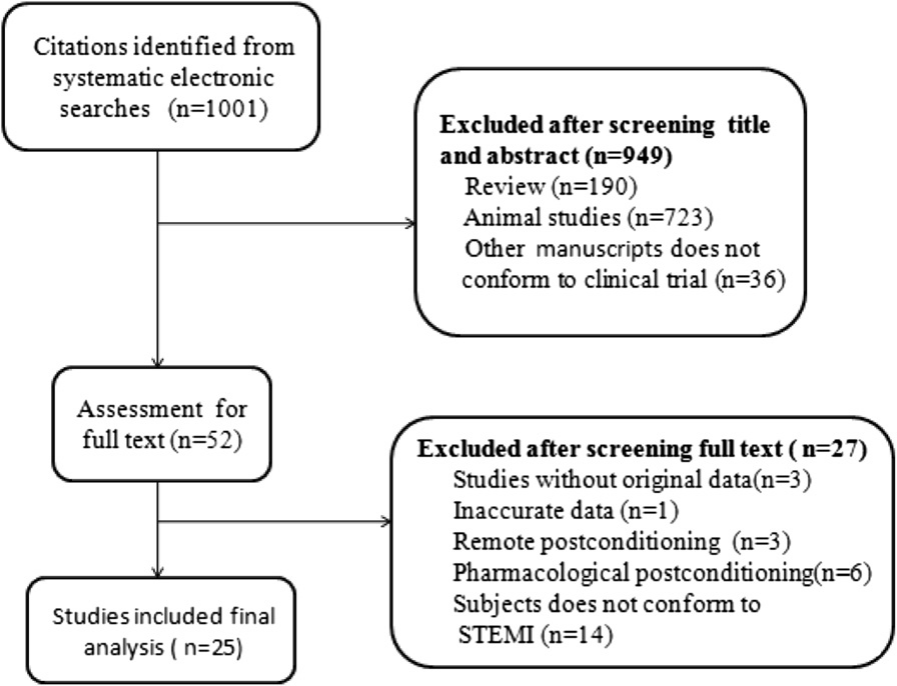
Flow diagram for the selection of articles

A total of 25 eligible publications were screened by the investigators. The studies that were excluded were as follows: (1) 949 manuscripts based on titles and abstracts and (2) 27 articles that either lacked original data [5, 13, 14], or were with inaccurate data [15], or involved remote postconditioning [16–18] and pharmacological postconditioning [19–24], or involved patients who were not suffering from STEMI and treated with PCI [6, 25–37]. The studies were excluded based on the full-text review. Twenty-five studies [7–11, 38–57] were included in this meta-analysis (Fig. 1).

### Study characteristics

Among the 2289 participants included in the meta-analysis, 1136 patients were in the postconditioning group and 1153 in the conventional care group. Characteristics of studies and patients are summarized in Tables 1 and 2.

**Table 1:** Characteristics of studies included in the meta-analysis

Author	Country	Year	Number (Poc/Con)	Male (%) (Poc/Con)	Age (y) (Poc/Con)	Dyslipidemia (%) (Poc/Con)	Diabetes (%) (Poc/Con)	Smokers (%) (Poc/Con)	Hypertension (%) (Poc/Con)
Staat [38]	France	2005	16/14	75/93	58/56	80/50	20/13	57/56	38/36
Ma [9]	China	2006	47/47	66/71	64/64	NR	38/45	NR	62/55
Ma [39]	China	2007	32/29	66/70	64/64	NR	38/45	NR	59/55
Yang [7]	China	2007	23/18	87/61	59/63	61/56	26/28	61/50	70/61
Laskey [40]	USA	2008	12/12	58/58	60/58	58/75	42/42	NR	75/83
Thibault [41]	France	2008	17/21	76/78	56/56	52/49	10/12	65/65	29/35
Lin1 [42]^a^	China	2010	25/26	84/65	59/63	56/42	24/27	56/50	52/54
Lin2 [42]^a^	China	2010	24/26	71/65	58/63	67/42	21/27	67/50	58/54
Lonborg [43, 44]^b^	Denmark	2010	59/59	69/74	61/62	46/41	7/7	61/49	37/32
Sorensson [45]	Sweden	2010	38/38	82/89	63/62	77/62	NR	26/29	16/29
Xue [46]	China	2010	23/20	95/94	54/62	16/24	21/29	63/71	37/71
Garcia [47]	USA	2011	22/21	86/76	61/55	73/71	5/19	23/43	73/71
Liu [48]	China	2011	30/34	73/68	59/59	NR	30/32	57/61	37/39
Freixa [10]	Spain	2012	39/40	84/72	59/60	44/35	23/17	51/62	49/50
Tarantini [49]	Italy	2012	37/38	85/85	60/60	51/49	18/3	67/77	59/49
Thuny [8]	France	2102	25/25	76/72	57/57	36/48	20/14	68/64	40/48
Zhao [50]	China	2012	30/32	97/87	57/62	31/17	13/27	69/77	50/67
Dwyer [51]	Canada	2013	50/52	88/89	57/57	36/29	6/14	44/44	42/33
Elzbieciak [52]	Poland	2013	18/21	67/86	60/58	61/86	22/24	67/52	78/91
Hahn [53]	Korea	2013	350/350	79/75	60/60	40/46	24/25	53/52	46/46
Mewton [54]	France	2013	25/25	76/72	57/57	NR	20/14	68/64	48/40
Sorensson [55]	Sweden	2013	33/35	85/89	63/62	NR	29/32	27/26	15/31
Dong [56]	China	2014	32/30	63/73	70/68	NR	34/37	41/50	72/63
Limalanathan [11]	Norway	2014	120/129	84/80	61/60	NR	4/2	49/54	29/25
Waltenberger [57]	Germany	2014	25/27	68/70	60/60	12/11	12/7	60/55	48/33

^a^Lonborg et al. published 2 articles on the same trial

^b^Lin et al. compared 60-s postconditioning with 30-s postconditioning and no postconditioning (routine) in this study

**Table 2:** Characteristics of studies included in the meta-analysis

Author	Country	Year	Chest pain	Elapsed time (m)	Culprit lesion	Protocal	Endpoint	Follow-up
Staat	France	2005	≤12 h	NR	LAD (38/43); RCA (62/57)	60″/60″ × 4	CKAUC;peak CK; blush grade; STR	3d
Ma	China	2006	≤12 h	395 ± 150/426 ± 150	LAD (49/53); LCX (23/17); RCA (28/30)	30″/30″ × 3	peak CK;peak CK-MB; WMSI	7d;2 m
Ma	China	2007	≤12 h	NR	LAD (53/52); LCX (22/17); RCA (25/31)	30″/30″ × 3	peak CK;peak CK-MB; WMSI	7d;2 m
Yang	China	2007	NR	312 ± 48/264 ± 42	LAD (65/61); LCX (9/6); RCA (26/33)	30″/30″ × 3	peakCK; CKAUC;IS; LVEF	3d;1w
Laskey	USA	2008	≤6 h	228 ± 43/222 ± 54	LAD (100/100); LCX (0); RCA (0)	90″/180″ × 2	peak CK; STR	NR
Thibault	France	2008	≤6 h	283 ± 82/297 ± 104	NR	60″/60″ × 4	CKAUC; TnI;IS; LVEF	6 m;12 m
Lin	China	2010	≤12 h	NR	LAD (64/62); LCX (8/8); RCA (28/30)	30″/30″ × 3	TNFα; LV EF (7d、1y); WMSI	7d;1y
Lin	China	2010	≤12 h	NR	LAD (54/62); LCX (8/77); RCA (38/31)	60″/60″ × 3	TNFα;LV EF (7d、1y); WMSI	7d;1y
Lonborg	Denmark	2010	≤12 h	241 ± 148.9/255 ± 196	LAD (44/39); LCX (8/19); RCA (47/42)	30″/30″ × 4	IS;IS/AAR;LVEF;peak TnT	3 m
Sorensson	Sweden	2010	≤6 h	165 ± 63.7/185 ± 87.41	LAD (37/37); LCX (11/3); RCA (53/61)	60″/60″ × 4	IS/AAR; TnTAUC; LVEF	6-9d
Xue	China	2010	≤12 h	4.1 ± 3.0/5.4 ± 3.7	LAD (42/59); LCX (0); RCA (58/41)	60″/60″ × 4	CK-MB, IS, LVEF, STR	7d
Garcia	USA	2011	≤12 h	4.5/4.4	LAD (36/24); LCX (23/10); RCA (41/67)	30″/30″ × 4	peakCK; CK-MB;LVEF	3.4y
Liu	China	2011	≤12 h	312 ± 102/324 ± 108	LAD (53/59); LCX (10/12); RCA (37/29)	30″/30″ × 3	peak CK; Peak CK-MB; WMSI;LVEF; blush grade;IS	7d
Freixa	Spain	2012	≤12 h	326 ± 180/330 ± 211	LAD (51/39); LCX (NR) RCA (45/47)	60″/60″ × 4	peak CK; Peak CK-MB; TnT; STR; IS (7d,6 m)	7d;6 m
Tarantini	Italy	2012	≤6 h	212 ± 85/194 ± 80	LAD (41/44); LCX (18/8); RCA (41/49)	60″/60″ × 4	peak TnI; LVEF;IS;	30d
Thuny	France	2102	≤12 h	289 ± 31/215 ± 20	LAD (56/56); LCX (0/8); RCA (44/36)	60″/60″ × 4	IS;peak CK	3d
Zhao	China	2012	≤12 h	309 ± 201/404 ± 191	LAD (53/53); LCX:NR; RCA:NR	60″/60″ × 4	LVEF; WMSI	1w;6 m
Dwyer	Canada	2013	≤6 h	150 ± 70/170 ± 84	LAD (50/46); LCX:(12/14); RCA:(38/40)	30″/30″ × 4	IS; AAR; PeakCK; LVEF	3d
Elzbieciak	Poland	2013	≤12 h	225.6 ± 139.4/317.6 ± 195.8	LAD (100/100); LCX (0) RCA (0)	60″/60″ × 4	IS; AAR; PeakCK-MB; peak TnT; LVEF;IS/AAR	3d;6 m
Hahn	Korea	2013	≤12 h	196 ± 51/195 ± 171	LAD (47/45); LCX:(11/11); RCA:(42/44)	60″/60″ × 4	peakCK-MB; STR; blush grade	30d
Mewton	France	2013	≤12 h	289 ± 31 /215 ± 20	LAD (56/56); LCX (0/8); RCA (44/36)	60″/60″ × 4	LVEF;IS;IS/AAR	4d
Sorensson	Sweden	2013	≤6 h	165 ± 51/180 ± 84	LAD (33/37); LCX (9/3); RCA (57/60)	60″/60″ × 4	IS; LVEF	12 m
Dong	China	2014	≤12 h	300 ± 90/294 ± 66	LAD (56/43); LCX (6/10); RCA (38/47)	30″/30″ × 3	blush grade; STR; CK- MB; TnT; LVEF	7d;30d
Limalanathan	Norway	2014	≤6 h	NR	LAD (46/51); LCX (13/9); RCA (41/41)	60″/60″ × 4	IS (CECMR); STR; TnT; LVEF	2d;4 m
Waltenberger	Germany	2014	≤6 h	NR	LAD (28/26); LCX (NR); RCA (64/74)	30″/30″ × 10	CKAUC;IS; LVEF	4d;4 m;12 m

NR,not related; LAD,left anterior descending branch; RCA,right coronary arterry; LCX,left circumflex artery; CK,creatine kinase; CKAUC,CK area under the curve; CK-MB,creatine kinase isoenzyme; STR,ST resolution; WMSI, wall motion score index; IS, infarct size; LVEF, left ventricular ejection fraction; TnI,troponin I; TNFα,tumor necrosis factor; AAR,area at risk; TnT, troponin T

The Poc protocol (cycles × ischemia/reperfusion in seconds) varied between studies, being 2 × 90″/180″ in 1 study, 10 × 30″/30″ in 1 study, 3 × 30″/30″ to 4 × 30″/30″ in 9 studies, and 3 × 60″/60″ to 4 × 60″/60″ in 14 studies. The follow-up in the trials varied from 3 days to 3.4 years. The relevant outcomes in all studies include markers of cardiac injury and left ventricular function (Table 2).

PCI was performed by direct stenting in some studies [7–9, 11, 38–42, 47–49, 52, 56]. However, in other studies [10, 43–46, 50, 51, 53, 55, 57], the choice of stent was left to the discretion of the operator. Balloon angioplasty or thrombus aspiration was also allowed if a stent could not be deployed or was considered harmful.

In the eligible studies, troponin levels were measured in eight studies [10, 11, 41, 44, 45, 49, 52, 56]. Eighteen [7, 9, 10, 38–42, 45–49, 51, 53, 54, 56, 57] studies contained data on peak or the area under the curve of CK or CK-MB. IS was measured by single-photon emission computed tomography (SPECT) or cardiovascular magnetic resonance (CMR) in eleven studies [7, 8, 10, 11, 41, 44, 46, 49, 51, 52, 57]. Global left ventricular function as determined by LVEF was measured in nine studies by echocardiography [7, 41, 42, 46, 47, 49–52], in eight studies by CMR [8, 11, 44, 45, 51, 54, 55, 57], and in one study by both echocardiography and CMR [10]. Regional left ventricular function was measured by WMSI in six studies [9, 39, 42, 46, 48, 50].

### Study quality

The analysis of the study quality in the 25 eligible studies is presented in Table 3. Baselines between the Poc group and the control group in all the studies were comparable. The measurement data was compared by the Student *t* test, and count data was compared by *χ*^2^ test between the two groups. In terms of quality, each of these studies would be graded level B according to the Cochrane Reviewer’s Handbook 4.2 for quality assessment of included RCTs.

**Table 3:** Quality assessments of studies included in the meta-analysis

Study	Year	Randomization	Blinded assessment	Dropout rate (%)	Similar baseline
Staat	2005	yes	no	unclear	yes
Ma	2006	yes	no	unclear	yes
Ma	2007	yes	no	unclear	yes
Yang	2007	yes	unclear	unclear	yes
Laskey	2008	yes	unclear	0	yes
Thibault	2008	yes	single-blind	0	yes
Lin1	2010	yes	no	unclear	yes
Lin2	2010	yes	no	unclear	yes
Lonborg	2010	yes	unclear	0.26	yes
Sorensson	2010	yes	no	0.15	yes
Xue	2010	yes	unclear	0	yes
Garcia	2011	yes	unclear	0	yes
Liu	2011	yes	unclear	0	yes
Freixa	2012	yes	no	0	yes
Tarantini	2012	yes	no	0.04	yes
Thuny	2102	yes	no	0.19	yes
Zhao	2012	yes	no	0.09	yes
Dwyer	2013	yes	unclear	0.23	yes
Elzbieciak	2013	yes	no	0	yes
Hahn	2013	yes	no	0.04	yes
Mewton	2013	yes	no	0	yes
Sorensson	2013	yes	no	0	yes
Dong	2014	yes	unclear	0	yes
Limalanathan	2014	yes	no	0.08	yes
Waltenberger	2014	yes	single-blind	0.13	yes

### Biomarkers of acute myocardial injury

**Fig. 2:**
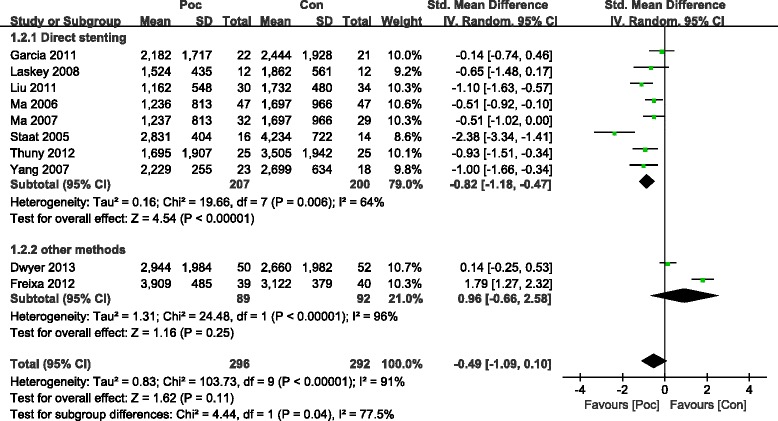
Effect of postconditioning on CK release during 72 h after PCI

**Fig. 3:**
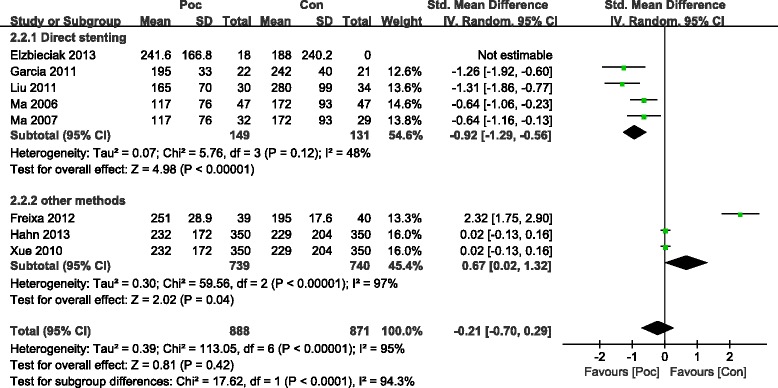
Effect of postconditioning on CK-MB release during 72 h after PCI

**Fig. 4:**
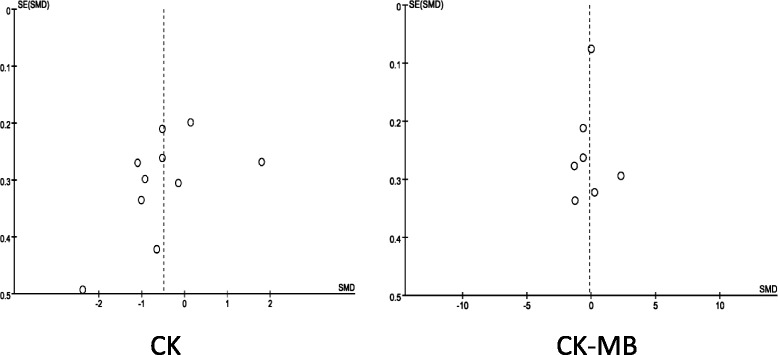
Funnel plot for the analysis of acute myocardial injury biomarkers

The commonly measured myocardial injury biochemical markers of acute myocardialinfarction (AMI) are CK and CK-MB, which are associated with infarct size. Compared with the Con group, the Poc group showed no apparent decrease in the level of peak CK after AMI [standard mean difference (SMD) = −0.49; 95 % confidence interval (CI), −1.09 to−0.1; I2 = 91 %; *P* =0.11). There was substantial between-study heterogeneity (Cochran Q test, *P* < 0.00001, *I*^2^ = 91 %). Subgroup analysis based on the method of PCI (PCI was performed only by direct stenting in some studies,and by other methods including direct stenting,balloon dilatation,and thrombus aspiration in rest of studies) showed that not only the decrease in CK became more significant but the heterogeneity also dropped with direct stenting (SMD = −0.82; 95 % CI, −1.18 to−0.47;*I*^2^ = 64 %;*P* < 0.00001) as compared with other methods (SMD = 0.96; 95 % CI, −0.66 to 2.58;*I*^2^ = 96 %;*P* =0.25) (Fig. 2). The CK-MB result was similar to those of CK (Fig. 3). The funnel plots with respect to the end point of CK and CK-MB showed no significant publication bias (Fig. 4). Publication bias measured by Egger’s test was not significant (*P* = 0.21, *P* = 0.68, respectively). These results showed that Poc can reduce ischemic necrosis of myocardium after acute infarction when patients received direct stenting.

### Myocardial infarct size measured by imaging

**Fig. 5:**
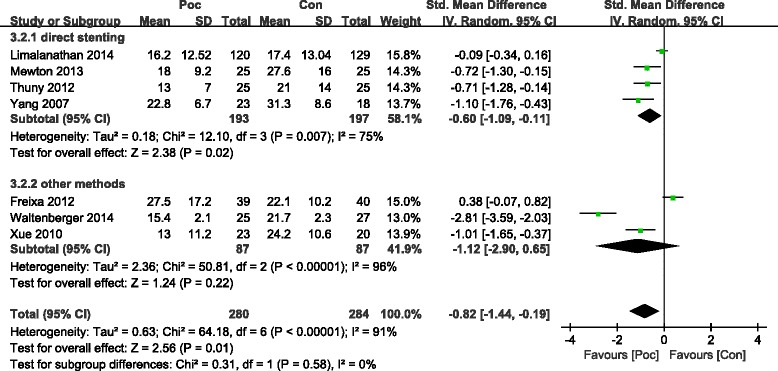
Effect of postconditioning on IS reduction during 7 days after PCI

**Fig. 6:**
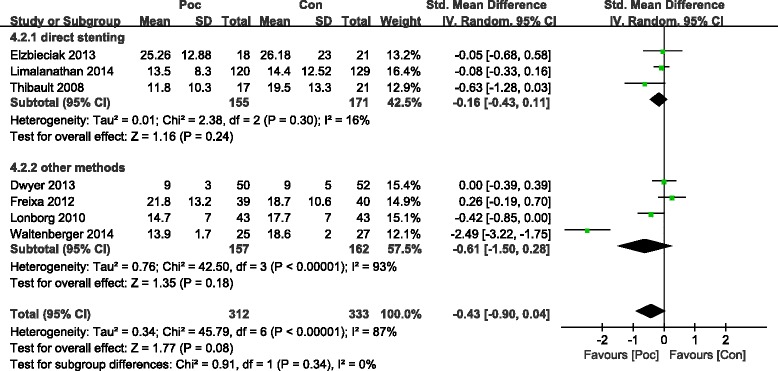
Effect of postconditioning on IS 4–12months after myocardial infarction

The meta-analysis of the studies demonstrated that the perfusion defect index on SPECT or CMR,an estimate of infarct size during 72 h after AMI, significantly reduced in the Poc group compared to the Con group. The pooled outcome of studies suggested a reduction in IS as measured by imaging (SMD = −0.82; 95 % CI, −1.44 to−0.19; *I*^2^ = 91 %; *P* =0.01). There was substantial between-study heterogeneity (Cochran Q test, *P* < 0.00001, *I*^2^ = 91 %) during 72 h after AMI. Subgroup analysis based on direct stenting showed that the heterogeneity dropped with direct stenting (SMD = −0.6; 95 % CI, −1.09 to−0.11;*I*^2^ = 75 %;*P* = 0.02) as compared with other methods (SMD = −1.12; 95 % CI, −2.9 to 0.65;*I*^2^ = 96 %; *P* =0.22) (Fig. 5). However, no significant difference was noted between the two groups more than 4 months after AMI (SMD = −0.43; 95 % CI, −0.9 to−0.04; *I*^2^ = 87 %; *P* = 0.08), while there was a trend toward the reduction of IS. Subgroup analysis based on direct stenting showed that the heterogeneity dropped with direct stenting (SMD = −0.16; 95 % CI, −0.43 to−0.11; *I*^2^ = 16 %; *P* = 0.24) as compared with other methods (SMD = −0.61; 95 % CI, −0.1.5 to 0.28; *I*^2^ = 93 %; *P* =0.18) (Fig. 6). No significant change in heterogeneity was noted when the studies were grouped based on geographic region.

### Cardiac function measures

**Fig. 7:**
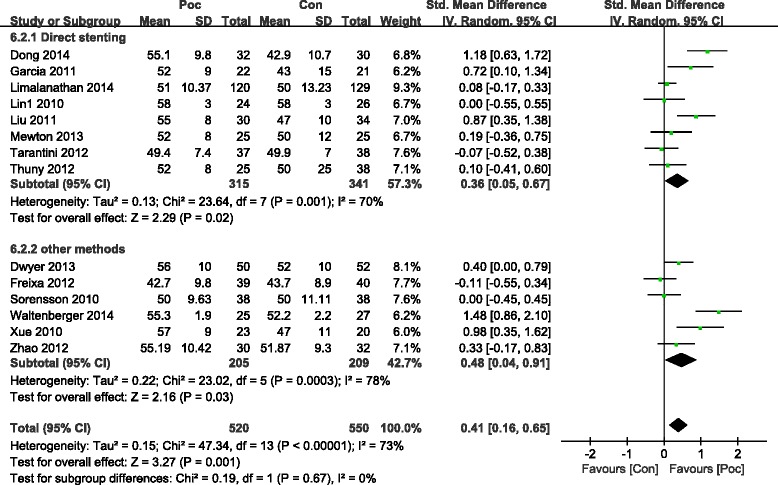
Effect of postconditioning on LV EF during 7 days after PCI

**Fig. 8:**
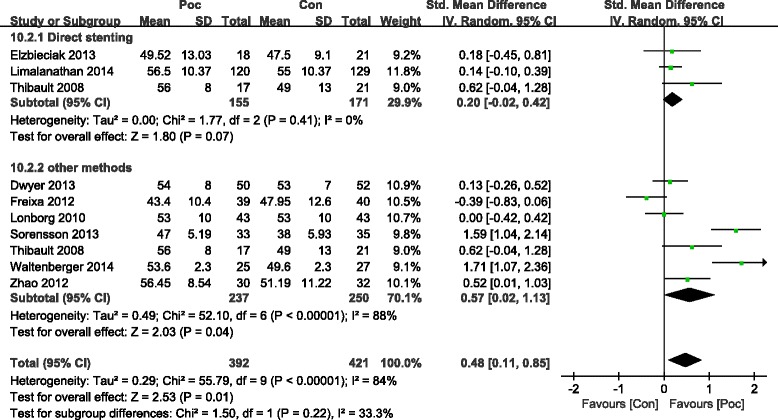
Effect of postconditioning on LV EF 4 months after myocardial infarction

**Fig. 9:**
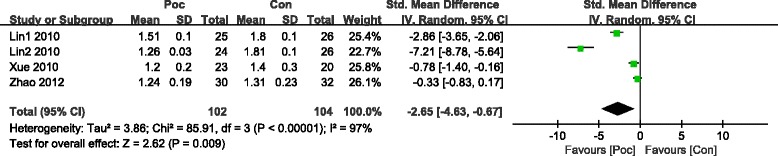
Effect of postconditioning on WMSI during 7 days after myocardial infarction

**Fig. 10:**
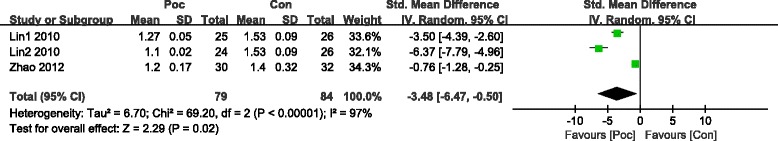
Effect of postconditioning on WMSI 6 months after myocardial infarction

LVEF was significantly improved in the Poc group in the short term within 7 days after AMI (SMD = 0.41; 95 % CI, 0.16 to 0.65 *I*^2^ = 73 %; *P* = 0.001) (Fig. 7). LVEF also improved in the Poc group than in the Con group over 4 months after AMI (SMD =0.48; 95 % CI, 0.11 to 0.85; *P* =0.01). However, subgroup analysis showed that improvement in LVEF was not significant. The heterogeneity dropped with the involvement of direct stenting (SMD =0.2; 95 % CI, −0.02 to 0.42; *I*^2^ = 0 %;*P* =0.07) as compared with other methods (SMD = 0.57; 95 % CI, 0.02 to 1.13;*I*^2^ = 88 %;*P* =0.04) (Fig. 8). Patients receiving Poc had a lower WMSI (a value of 1 is normal segmental motion and higher values indicate poorer contraction) than those receiving usual primary PCI in both the short term within 7 days (SMD = −2.65; 95 % CI,−4.63 to−0.67; *P* = 0.009) (Fig. 9) and the long term over 6 months (SMD = −3.48; 95 % CI,−6.47 to−0.5; *P* = 0.02) (Fig. 10).

## Discussion

The current meta-analysis included data from 25 randomized trials involving 2289 participants. The results gave rise to a view that postconditioning following PCI induced by transient coronary ischemia in STEMI patients may reduce myocardial injury biomarkers and improve cardiac function, compared with the usual care group. This cardioprotection was more apparent when direct stenting was performed in PCI compared with other methods of PCI, including balloon angioplasty and thrombus aspiration. The lack of substantial effect of Poc when PCI was completed by angioplasty or thrombus aspiration was most likely due to inadequate revascularization. However, despite the trend in IS reduction, no significant decrease was noted in IS over 4 months after AMI.

Reperfusion therapy is an effective therapeutic approach during the early stage of STEMI patients to prevent heart failure and other cardiovascular events. Many studies have verified that immediate reperfusion is critical to rescue the ischemic myocardium. However, reperfusion has the potential exacerbation of myocardium injury, including myocardial stunning, no reflow, and ventricular arrhythmias [58–60]. Therefore, attenuating reperfusion injury has become an urgent challenge for salvaging myocardium during reperfusion in STEMI patients. Poc performed during angioplasty is technically simple and safe, for cycles of “ischemia/reperfusion” can be easily achieved by repeatedly deflating and inflating the balloon in the culprit artery. A number of studies [7–9, 38–44, 46, 48, 50, 54, 56, 57] described salutary effects of Poc on ischemic/reperfusion injury. In contrast, other studies [10, 11, 45, 47, 49, 51–53, 55] show negative effect of postconditioning, even harmful for myocardium salvage. These studies present high heterogeneity of result due to the difference of Poc protocol, measurement, culprit artery, chest pain elapsed time, endpoints, and the type of PCI. For example, IS can be measured by SPECT or CMR, assessed by percentage of the area at risk, a percentage of the left ventricular mass,or in grams. A pooled analysis of RCTs has shown that involvement of the left anterior descending (LAD) is one of the strongest predictors of IS [61]. This study revealed that postconditioning presents cardioprotection in patients with STEMI, especially in whom PCI was performed by direct stenting. The reason may be that reperfusion is more adequate by direct stenting than other methods. Appropriate trials are needed to answer this question. Eleven studies performed follow-up from 3 months to 3.4 year [10, 11, 41, 42, 44, 47, 50–52, 55, 57]. The short-term (within 7 days after PCI) beneficial effect in the Poc group included reduced biomarkers of myocardium injury, reduced IS measured by imaging,and the left ventricular function assessed by LVEF or WMSI. Unfortunately, the remarkable decrease of IS did not appear after the long-term follow up. In both short- or long-term follow-up, the global left ventricular function as determined by LVEF improved. Although the analysis suggest that regional left ventricular function assessed by WMSI showed positive result in both short- and long-term follow-up,only several studies cover WMSI. Due to the limited sample size, the results should not be considered conclusive.

Poc could improve myocardial reperfusion in patients with ST-elevation AMI undergoing PCI by reducing no reflow. However, the mechanisms of Poc are not clear. Previos study revealed that high concentrations of inorganic phosphate, reactive oxygen species, and reactive nitrogen species are all present during myocardial ischemia and during reperfusion [62]. Due to the importance of oxidative stress and inflammation in atherosclerotic plaques development and Cardiovascular disease progression, therapeutic of antioxidant seems to be very important [63]. So the cardioprotective effect of Poc partly depend on antioxidant and anti-inflammatory.

Moreover, the question is whether different protocols have different cardioprotective effects. Many different protocols of postconditioning existed, such as 60 s × 3circles, 60 s × 4circles, 30 s × 4circles, and so on, used in available studies. However, only one study tested the hypothesis that postconditioning of 60 s × 3 was more protective than postconditioning of 30 s × 3 [42]. Therefore, additional trials of a large scale are needed to determine the optimal protocol. It is also reported that ischemic postconditioning reduced infarct size in normotensive but not hypertensive rat hearts [64, 65]. Experimental animal data suggest that the presence of diabetes and related conditions, such as obesity and metabolic syndrome, may affect the cardioprotective efficacy of both ischemic and pharmacologic postconditioning. Przyklenk et al. [66] found that ischemic postconditioning cannot play a protective role in reducing infarct size by isolated perfused heart of murine model compared with the normoglycemic heart. In a retrospective analysis, postconditioning the human heart by multiple balloon inflations failed to reduce irreversible injury in patients above the age of 65 years [67]. Yellon and colleagues demonstrated a decline in the effectiveness of RISK pathway signaling with age [68]. They suggested that cardioprotection associated with postconditioning may be affected by age, comorbidities, medications, and the method selection of PCI. Therefore, future strategies will need to focus on the quality of reperfusion. Appropriate trial design is required to provide clearer answers.

### Limitations

Compared with previous studies, the analysis of the present study involves a comprehensive literature search including a large number of relevant studies. Postconditioning was associated with a reduction of infarct size as determined by biochemical quantification and imaging. Cardiac function was assessed by LVEF and WMSI. In this meta-analysis, the long-term (over than 3 month) effect of postconditioning was analyzed as well as the short-term effect. Besides, method selection of PCI was based on subgroup analysis. However, the limitations inherent to the studies contained in the analysis impact the present-study results, such as small sample size, heterogeneity between studies, and risk of performance bias.

## Conclusion

Ischemic postconditioning during PCI in STEMI seems to be superior to the conventional PCI alone in reducing acute myocardial injury, infarction size, and left ventricular function including global and regional function, especially in patients who have received direct stenting in PCI. Given the limitations of the current available evidence, additional data involving potential risk factors of restricting postconditioning from large RCTs are needed.
